# Primary intraosseous meningioma: an osteosclerotic bone tumour mimicking malignancy

**DOI:** 10.1186/s13569-016-0054-2

**Published:** 2016-08-14

**Authors:** M. Vlychou, Y. Inagaki, R. Stacey, N. A. Athanasou

**Affiliations:** 1 Nuffield Department of Orthopaedics, Rheumatology and Musculoskeletal Sciences, University of Oxford, Nuffield Orthopaedic Centre Windmill Road, Oxford, OX3 7HE UK; 2 Department of Neurosurgery, John Radcliffe Hospital, Oxford University Hospitals, Headley Way, Oxford, OX3 9DU UK

**Keywords:** Intraosseous, Meningioma, Skull, Calvarium

## Abstract

**Background:**

Sclerotic tumours of the calvarial bones are rare and may be due to primary and secondary bone tumours as well as extradural tumours of meningeal origin.

**Case presentation:**

We report a case of primary intraosseous meningioma (PIM) which arose in the frontal bone of a 63 year old woman who complained of progressive pain and thickening of the right skull. Radiology showed a large osteosclerotic lesion in the right frontal bone. Histology showed an intraosseous lesion containing dense fibrous tissue in which there were scattered cells that expressed epithelial membrane antigen and progesterone receptor. The tumour was partially resected and 3 years after operation has not recurred.

**Conclusions:**

PIM is a rare tumour which needs to be distinguished from primary/secondary osteosclerotic calvarial bone tumours.

## Background

Primary tumours and tumour-like conditions of the cranial vault are rare in both children and adults [1–3]. These lesions usually present with non-specific pain and swelling and radiologically may be osteosclerotic, osteolytic or show a mixed picture of sclerosis and lysis. The differential diagnosis of these lesions is wide and includes not only primary and secondary bone tumours but also extradural tumours of meningeal origin that can involve or arise in skull bones [1, 3, 4]. We report a case of a primary intraosseous meningioma (PIM), which was markedly osteosclerotic and difficult to distinguish histologically and radiologically from a malignant primary/secondary bone tumour. The nature of this rare lesion is discussed and the differential diagnosis of osteosclerotic calvarial tumours reviewed.

## Case report

A sixty-three year old woman presented with progressive pain and thickening of the right skull frontal bone. Physical examination revealed a swelling of the right frontal region about 12 cm in diameter; the lesion was not adherent to the overlying skin and was not mobile or tender. The patient had no neurologic deficit but she complained of a sensation of pressure in the right eye, occasionally feeling unable to open it. Laboratory studies were unremarkable.

**Fig. 1:**
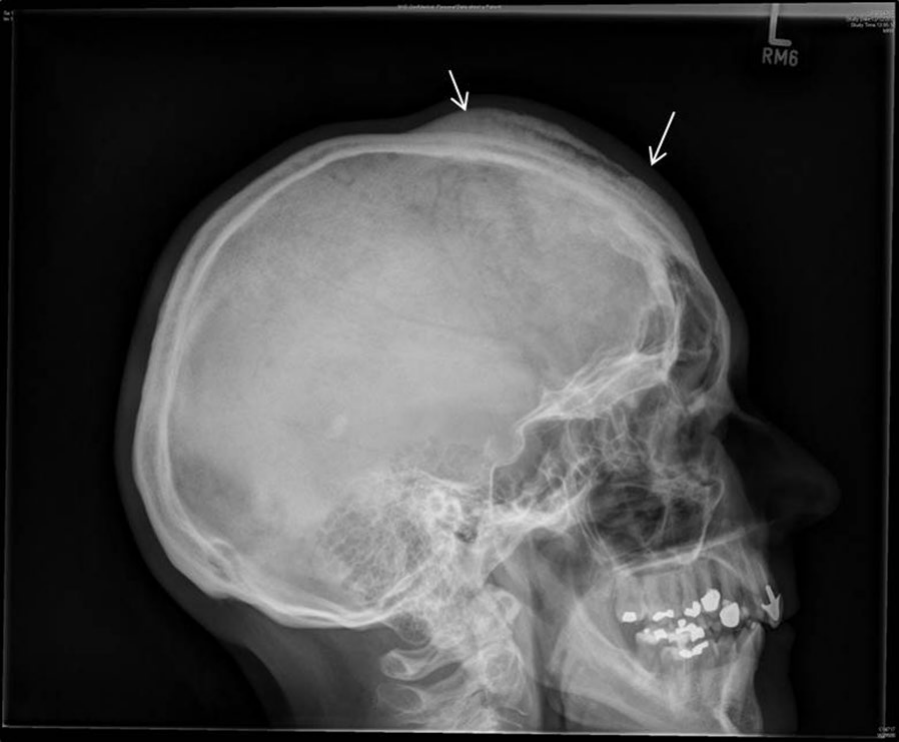
Lateral X-ray of the skull shows an extensive osseous lesion of the diploe with periosteal new bone formation (*arrows*) along the external table of the anterior skull

**Fig. 2:**
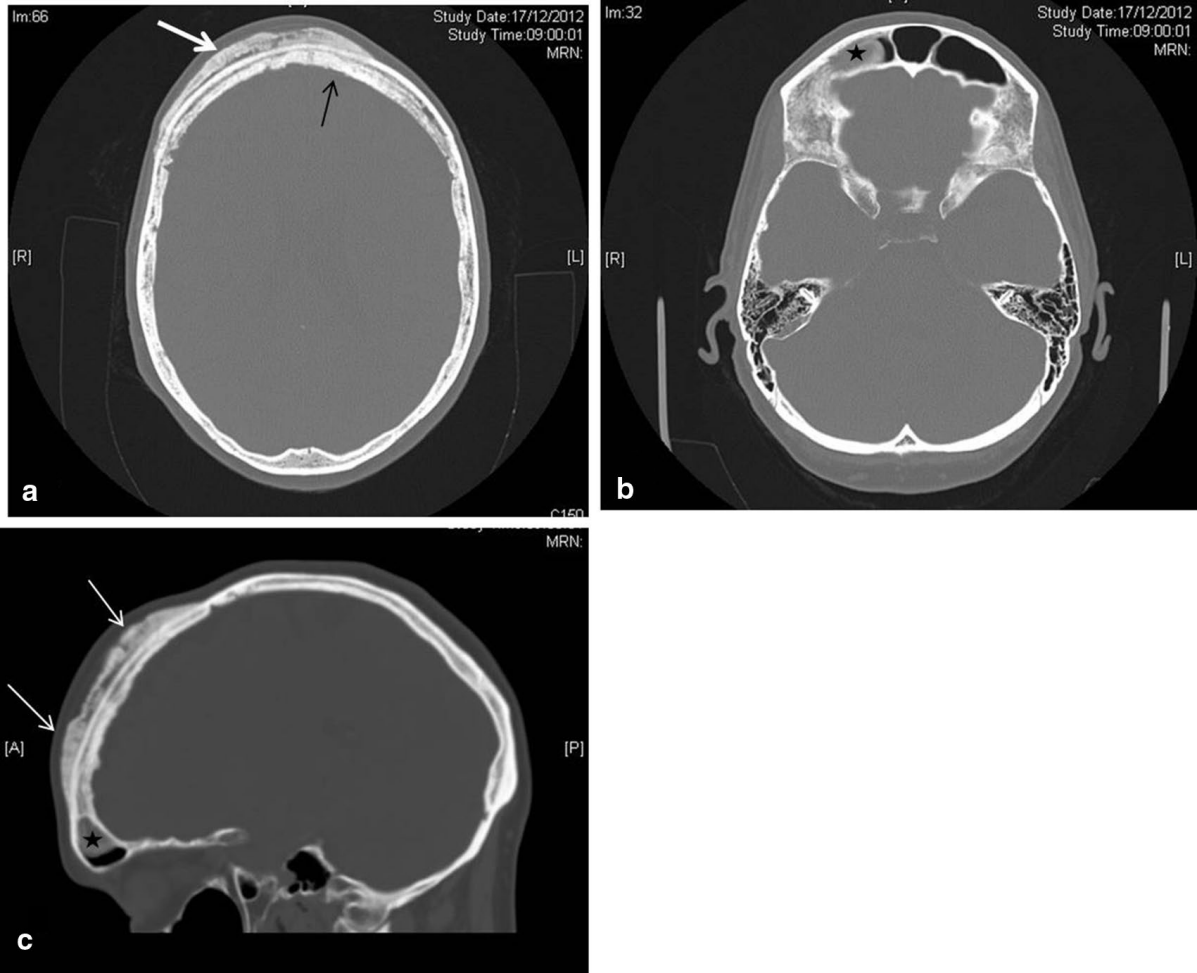
**a** CT axial image shows a primarily sclerotic lesion involving the diploe (*white arrow*) extending across the midline (*black arrow*). **b** There is a polypoid mass in the right frontal sinus (*asterisk*). **c** Sagittal reconstruction shows the lesion (*white arrows*) and the polypoid mass (*asterisk*)

**Fig. 3:**
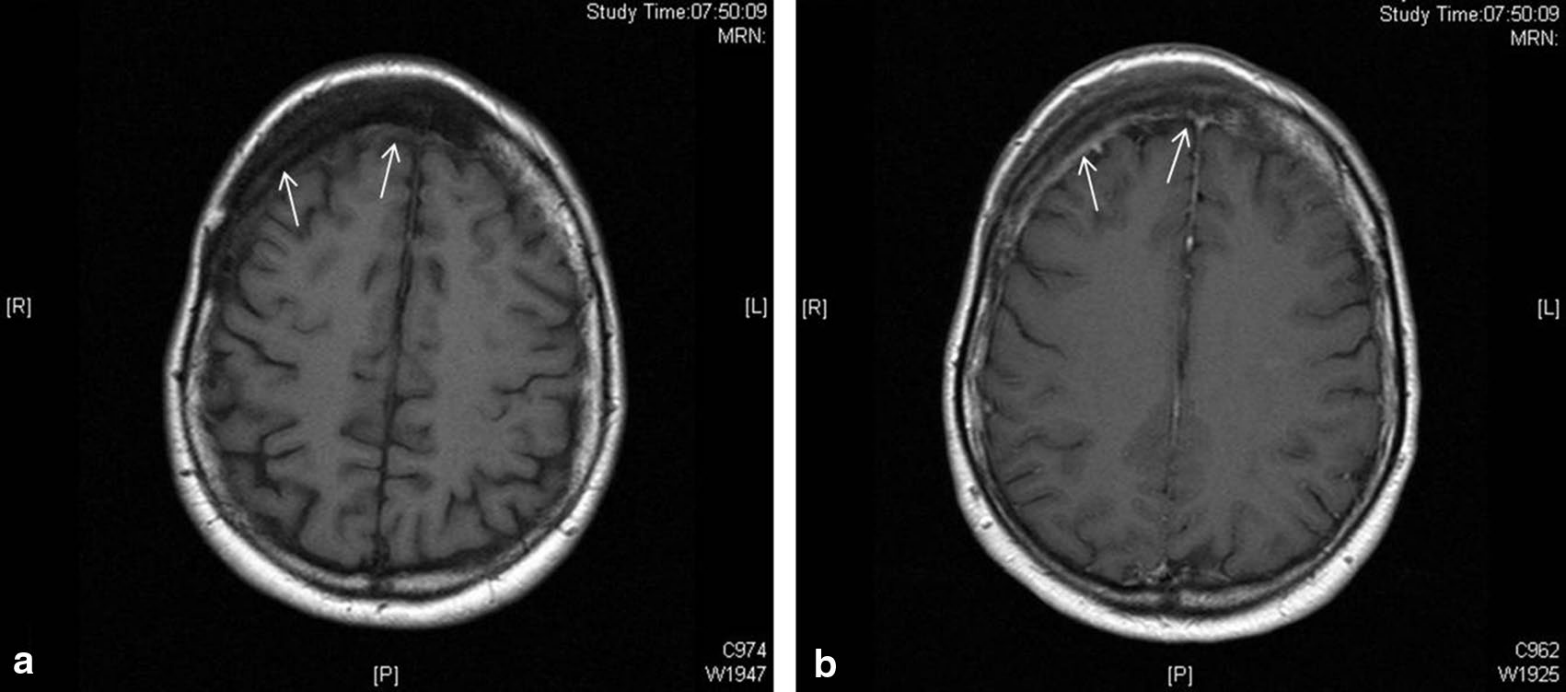
**a** Axial T1-weighted MR image shows thickening of the right frontal part of the diploe (*arrows*) with an underlying extensive low signal osseous lesion. **b** Axial T1-weighted MR image post gadolinium shows minimal enhancement of the periosteal part of the lesion (*arrows*). There is no evidence of abnormality related to the brain parenchyma

Plain x-ray films revealed a large, sclerotic lesion involving the right side of the frontal bone of the skull, extending to the unilateral frontal sinus (Fig. 1). Further imaging with CT scan showed a diffuse sclerotic right-sided frontal lesion measuring approximately 9.5 × 12 × 1.5 cm involving the diploe with extensive mature periosteal new bone formation along the external table of the right frontal bone (Fig. 2a–c). The lesion extended across the midline and into the right frontal sinus. An MRI scan confirmed the osseous nature of the tumour and the absence of infiltration of the brain parenchyma (Fig. 3a, b).

After biopsy of the lesion for histological diagnosis (see below), follow-up imaging showed progressive periosteal bone formation on the external table of the calvarium. Four months after the biopsy, a right frontal craniotomy with mesh cranioplasty was performed with resection of the calvarial tumour but not the mass in the frontal sinus. The patient made a good post-operative recovery and has remained clinically well with follow up imaging over 3 years showing that the residual tumour has not increased in size.

**Fig. 4:**
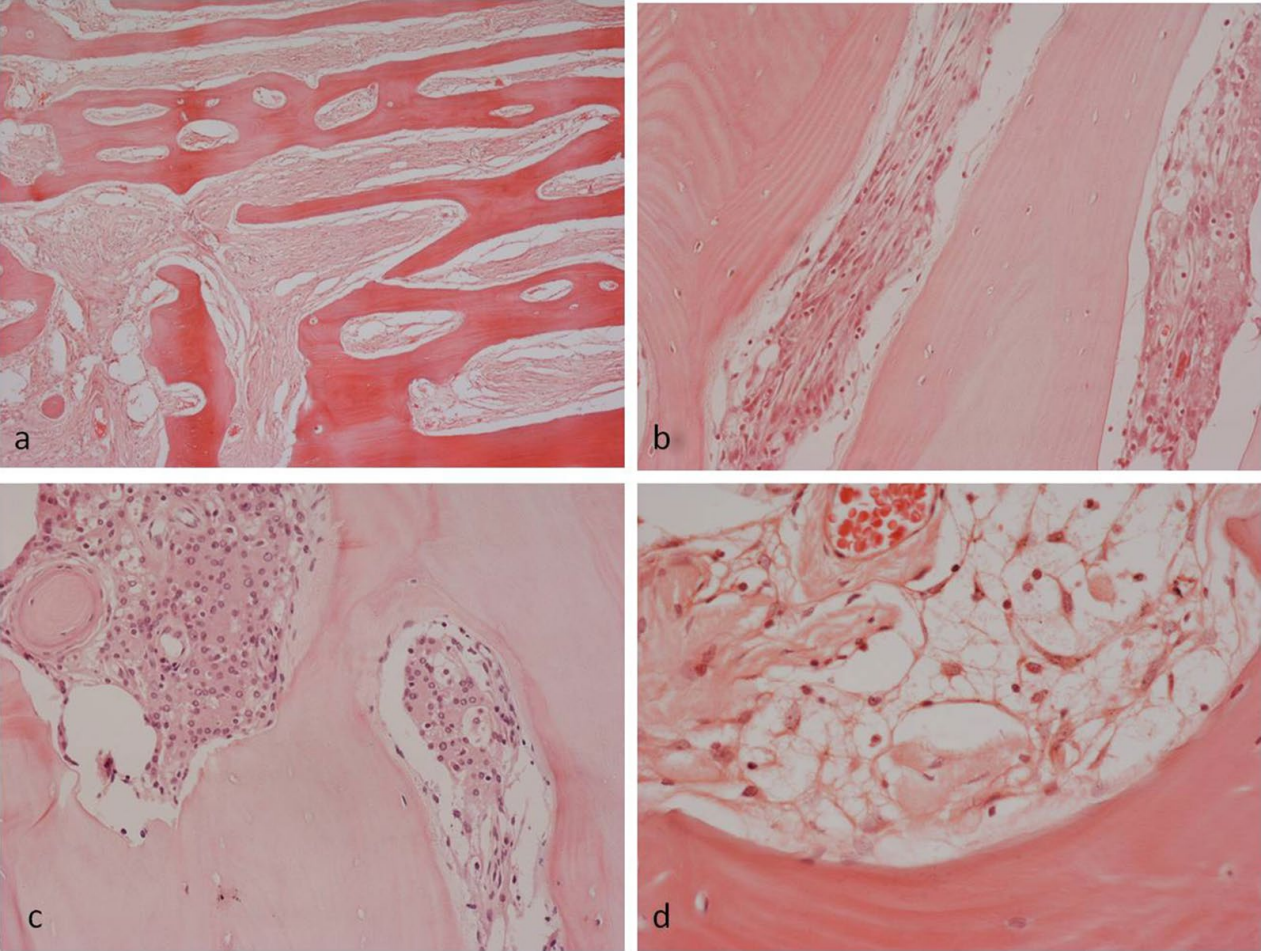
Histopathological features of PIM showing **a** extensive organised reactive bone formation within the lesion. Intertrabecular fibrous tissue contains **b** a proliferation of cells with spindle-shaped and vesicular nuclei; **c** scattered meningotheliomatous whorled collections of cells; **d** occasional cells with clear, vacuolated cytoplasm

**Fig. 5:**
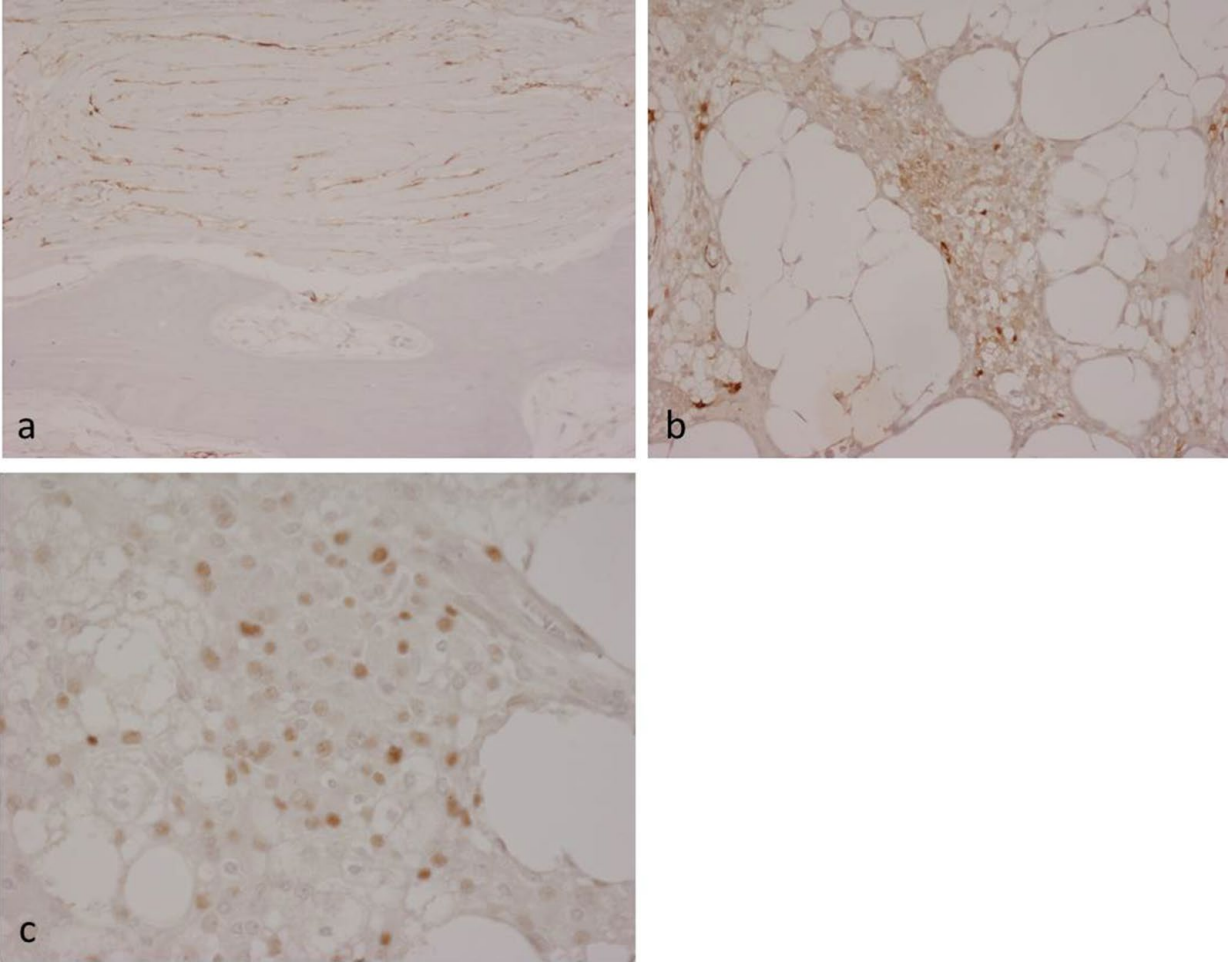
Immunohistochemistry of PIM showing lesional cell expression of **a** EMA in fibrotic areas and **b** EMA and **c** PR in meningotheliomatous areas

Histopathology of the fixed, decalcified biopsy and resection specimens showed similar features. There was abundant thickened organised reactive woven and lamellar bone with numerous thickened bone trabeculae separated by dense collagenous fibrous tissue containing scattered cells with wavy, spindle-shaped or ovoid vesicular nuclei between bone trabeculae and in Haversian channels (Fig. 4a, b). There were also numerous scattered cells with vacuolated cytoplasm and focally small meningotheliomatous whorls of cells with vesicular nuclei in fat and fibrous tissue (Fig. 4c, d). The tumour involved cortical and medullary bone and the dura but had not spread into superficial soft tissue beyond the periosteum. Immunohistochemistry showed that the lesional cells strongly expressed epithelial membrane antigen (EMA), vimentin and progesterone receptor (PR) (Fig. 5a–c). A few cells reacted for CD99 and podoplanin. There was no staining for estrogen receptor, CD45, CD68, CD10, chromogranin, Melan A, HMB45, CD34, CDK4, *SATB2*, desmin, smooth muscle/muscle actin, GFAP, NSE, cytokeratin (CK7−, CK20−), Brachyury, S100, DMP-1 CD31, CD34 or factor 8. There was a low Ki-67 proliferation index. The morphological features and strong expression of EMA and PR were considered to be consistent with the diagnosis of a partly fibroblastic intraosseous meningioma. (WHO Grade I).

## Discussion

Meningiomas arise from meningothelial cells that are found in the arachnoid membrane and line arachnoid villi associated with intradural venous sinuses and their tributaries [5, 6]. Meningioma is the most common primary brain tumour, comprising 20–30 % of all primary intracranial neoplasms. Most meningiomas are benign but there is considerable variation in clinical presentation, histological appearance, aggressive behavior and recurrence rate following surgical treatment. Meningiomas arise mainly in the supratentorial compartment, especially along the dural venous sinuses, particularly the superior sagittal sinus, over the cerebral convexities and in the region of the falx cerebri, although they can develop anywhere in the brain as well as in the spine (12 % of cases). Although the vast majority of meningiomas are attached to the dura and are intracranial tumours, they can develop in extracranial sites with primary extradural meningiomas accounting for less than 2 % of all meningiomas [7–9]; PIM, the rarest form of meningioma, is a subset of primary extradural meningioma.

Most PIMs involve the calvaria, particularly the frontoparietal and orbital regions [4, 7–9]. Primary extradural meningiomas, including PIMs, occur with approximately the same frequency in both genders; they have a relatively wide age range, occuring predominantly in later life, although they can be encountered in younger patients (especially during the second decade). Most patients with calvarial PIMs typically present, as in our case, with a slow-growing swelling that may or may not be painful. Neurological complications occur when the lesion extends through the inner table and compresses intracranial structures. Although most calvarial meningiomas are benign they undergo malignant change more commonly than intracranial meningiomas [7, 9]. Bone remodeling and calvarial thickening is frequently seen in PIMs as well as other meningiomas with osteosclerosis occurring more commonly than osteolysis. Conventional radiographs may show an osteosclerotic or osteolytic tumour. CT with bone windows may show cortical destruction and both intraosseous and extraosseous extension of the tumour [9–14]. MR imaging is necessary for the evaluation of the soft tissue component and extradural extension. PIMs need to be distinguished radiologically from osseous spread of meningiomas which have arisen intracranially as histological appearances can be similar. An extracalvarial meningioma that erodes the inner table of the skull with dural reaction and/or invasion is generally classified as being a primary extradural lesion. Lang et al. [8] classified primary extradural meningiomas into three types: type I, a purely extracalvarial tumour; type 2, a purely calvarial tumour; type 3 a calvarial tumour with extracranial extension (as in our case).

The pathogenesis of PIM is controversial. It has been suggested that PIMs may develop by entrapment of arachnoid cap cells in cranial sutures during pre/postnatal development of the skull or during delivery [7–9]. It has also been proposed that arachnoid cap cells may become entrapped in fracture sites after trauma. However, only 8 % of PIMs are associated with a cranial suture and only a small percentage of patients give a history of trauma prior to tumour development. In our case, there was no history of trauma or radiological evidence of an old skull fracture. The dura of the meninges is of mesodermal origin and the leptomeninges are mainly of neural crest (ectodermal) origin; it has been suggested that proliferation of pluripotent embryonal precursor cells in bone, capable of meningothelial differentiation, could account for the development of PIMs.

Calvarial bone tumours are rare, and meningioma involving skull bones, primary and secondary bone tumours and tumour-like lesions need to be considered in the differential diagnosis, particularly when, as in our case, the tumour is a large osteosclerotic lesion. Primary bone-forming tumours, such as osteoma, osteoblastoma, osteosarcoma and fibrous dysplasia (which commonly involves the skull and craniofacial bones) can arise in the frontal and other skull bones [15–21]. Cartilage tumours, notably chondromyoxid fibroma [22, 23], as well as metastatic cancer, plasmacytoma, Langerhans cell histiocytosis Paget’s disease, chronic osteomyelitis, have also been noted [1–3, 24–27]. Some of these lesions, as well as giant cell tumour, hemangioma, epidermoid cyst and aneurysmal bone cyst, may present as osteolytic tumours [28, 29]. PIMs exhibit similar morphological features to intracranial meningiomas [5, 6, 9, 30]. The diagnosis of meningioma can be challenging and this lesion can be mistaken histologically for primary and secondary osteosclerotic bone tumours [31–33]. In our case expression of EMA and PR was very useful in making the diagnosis of PIM and distinguishing this lesion from a primary or secondary bone tumour.

In symptomatic PIMs, total removal of the tumour followed by cranial reconstruction is the treatment of choice. If there is involvement of critical structures within sinuses or the skull base, it is recommended that subtotal resection is carried out and the status of the residual tumour be followed radiologically [7–9, 30, 33]. In our case, the tumour was large and symptomatic; the possibility of malignancy could not be excluded with confidence. Partial excision of the tumour was undertaken and the patient has remained well with no progression of the lesion. Adjuvant radiation therapy has been recommended in patients who have residual lesions that are symptomatic and/or show evidence of progression.

## Conclusion

PIM is a rare tumour which needs to be considered in the differential diagnosis of an osteosclerotic calvarial bone tumour. PIMs resemble intracranial meningiomas histologically and express EMA and PR. CT and MRI are useful in preoperative radiological assessment of PIMs which can be treated successfully by surgical resection.

## Abbreviations

PIM: primary intraosseous meningioma
EMA: epithelial membrane antigen
PR: progesterone receptor
WHO: World Health Organization
CT: computed tomography
MRI: magnetic resonance imaging
